# An Effective Dense Co-Attention Networks for Visual Question Answering

**DOI:** 10.3390/s20174897

**Published:** 2020-08-30

**Authors:** Shirong He, Dezhi Han

**Affiliations:** College of Information Engineering, Shanghai Maritime University, Shanghai 201306, China; 201830310085@stu.shmtu.edu.cn

**Keywords:** visual question answering, dense co-attention network, Bi-LSTM, deep learning, natural language processing, computer vision

## Abstract

At present, the state-of-the-art approaches of Visual Question Answering (VQA) mainly use the co-attention model to relate each visual object with text objects, which can achieve the coarse interactions between multimodalities. However, they ignore the dense self-attention within question modality. In order to solve this problem and improve the accuracy of VQA tasks, in the present paper, an effective Dense Co-Attention Networks (DCAN) is proposed. First, to better capture the relationship between words that are relatively far apart and make the extracted semantics more robust, the Bidirectional Long Short-Term Memory (Bi-LSTM) neural network is introduced to encode questions and answers; second, to realize the fine-grained interactions between the question words and image regions, a dense multimodal co-attention model is proposed. The model’s basic components include the self-attention unit and the guided-attention unit, which are cascaded in depth to form a hierarchical structure. The experimental results on the VQA-v2 dataset show that DCAN has obvious performance advantages, which makes VQA applicable to a wider range of AI scenarios.

## 1. Introduction

Visual Question Answering (VQA) is a multimodal research task that aims to answer questions related to the given image. Compared with other multimodal learning tasks (e.g., visual description [1], visual grounding [2,3,4], multimodal embedding learning [5,6,7,8]), VQA requires a fine-grained semantic understanding of both visual and textual content to predict the correct natural language answer. Therefore, VQA has recently emerged as an extremely challenging task and drawn considerable attention from researchers.

The attention mechanism is significant progress in VQA, which is essentially similar to the attention mechanism of human vision and aims to select what is more critical to the current objectives from a wide range of information. The attention mechanism has played an important role in various tasks concerning artificial intelligence since it is proposed in Reference [9], making VQA applied in intelligent robots [10], driverless cars, and navigation for visually impaired people [11], early childhood education, sensor technology, and other fields [12]. For example, VQA can be applied to an environment that is monitored and perceived by sensors. The sensors collect environment information in real time, such as temperature, pressure, smell, or the exact location of an object. Then the VQA network takes three kinds of information as input to predict answers: the image of the current scene, the information collected by the sensors, and questions. Moreover, the attention mechanism improves the performance of unimodal tasks, such as vision [13], language [14,15], and so on [16]. In addition to the visual attention model, researchers had also proposed a co-attention model [17,18], which can learn visual attention and text attention simultaneously. However, it can only learn the coarse interactions between multimodalities, and can not infer the relationship between an image and the keywords of a question. To solve this problem, References [19,20] proposed more comprehensive co-attention models to capture the complete interactions between words and regions, and further expanded them to form a deep co-attention model. However, these two models only have a slight performance improvement compared with the shallow models. Inspired by Transformer [21] and References [22,23], two deep co-attention networks (e.g., MLIN [24], MCAN [25]) have been proposed, which significantly outperform the previous approaches.

Despite the excellent performance of the current co-attention models, the interaction within question modality is insufficient. Complex relationships between words can be learned when two parallel question self-attention units are used to extract question features, which contributes to understanding the image and provides complementary and vital information to the image, thus facilitating more accurate multimodal reasoning. Therefore, we propose an effective Dense Co-Attention Networks (DCAN), the core of which is the Dense Co-Attention (DCA) layers stacked in depth. Each DCA layer consists of two parallel question self-attention units, an image self-attention unit, and a guided-attention unit. The self-attention unit aims to carry out intra-modal interactions, while the guided-attention unit is used to realize the inter-modal interactions between the image regions and question words. Compared with the single-layer self-attention unit in MCAN, two parallel question self-attention units can extract more fine-grained question features. When the question features are used to guide the image, more accurate image features can be obtained. Experimental results on the benchmark VQA-v2 dataset [26] demonstrate our model is reasonable and effective. Additionally, compared with LSTM, Bidirectional Long Short-Term Memory (Bi-LSTM) [27] neural network can theoretically better capture the bidirectional semantic dependencies. It is beneficial to feature extraction of the dense co-attention learning module. Therefore, Bi-LSTM is employed to encode questions. Finally, the ablation studies under one DCA layer proved that Bi-LSTM is slightly better than LSTM.

In summary, the main contributions of this paper are as follows:

- An improved multimodal co-attention model is proposed by stacking the self-attention unit and the guided-attention unit. It can not only describe the interactions between multimodalities in a more effective way but also take account of the dense self-attention in each modality. Compared with the existing scheme MCAN, DCAN achieves higher precision.
- Ablation studies on VQA-v2 are conducted to explain the effectiveness of DCAN. The qualitative evaluation results demonstrate how it generates reasonable attention to questions and images.

The rest of this paper is organized as follows—the related work is introduced in Section 2. Then co-attention layer is described in Section 3. The overall architecture of dense multimodal co-attention networks is presented in Section 4. The ablation studies are given in Section 5. The conclusion is provided in the last section.

## 2. Related Work

### 2.1. Attention-Based Vqa Model

When looking at an image, the focus is necessarily on a certain part of the image. In other words, when shifting eyes to another place, attention is also shifting along with the movement of the eyes. In this sense, when people notice a target or scene, the distribution of attention within the target or at each spatial location in the scene is different. With reference to the way the human brain processes information, the attention mechanism is introduced in deep learning, which can quickly select useful information from large amounts of data. A series of methods based on the attention mechanism came into being, but these methods are not the same.

Reference [17] had achieved great success in the VQA task. The word features are aggregated through the image-guided attention mechanism, and the image features of all regions are aggregated into global image embedding. This co-attention framework uses concatenation and average pooling to merge all components. Shih et al. [28] proposed a method of learning to answer visual questions by selecting image regions related to text query, which maps the text queries and visual features of different regions into a shared space. Fukui et al. [29] used multiple attention layers or generated multiple attention maps to realize multi-step reasoning or multiple attention on images. Li et al. [30] extracted the bounding boxes in the image and scored each bounding box according to the text features. Anderson et al. [31] proposed to combine bottom-up and top-down attention to calculate attention at the level of objects and other prominent image regions. It expands the number of object classes from 200 to 1600, and Visual Genome [32] is utilized for data augmentation. Kim et al. [19] extended the attention network, in which low-rank bilinear pooling is used to extract joint representations of multimodal inputs. Reference [20] showed that the dense and bidirectional co-attention mechanism between two modalities contributes to improving prediction accuracy. In Reference [25], a deep modular co-attention network was proposed, which associates the keywords in questions with the critical regions in images.

### 2.2. Multimodal Feature Fusion

Multimodal feature fusion [33,34] refers to the fusion of visual features from images and text features from questions. The question is that the abstraction of the real world takes the form of texts featuring a high semantic level. In comparison, the image exists in the form of pixels, and the aggregation of pixels produces semantics. The image thus has a relatively low semantic level but contains rich information to reflect the real world. Therefore, there is an inevitable semantic gap between images and questions, which requires a complex interaction between image features and question features.

At present, the fusion methods include the method based on linear fusion and the method based on bilinear pooling. The former includes feature connection and element multiplication and other linear operations. The latter is expressed as the outer product of two vectors. However, the dimension of the feature obtained by the ordinary exterior product is the square of the original feature’s size, making the subsequent classification model large. Therefore, the academic community has made various improvements to the bilinear pooling method, which significantly reduces the dimension of features. Kim et al. [35] put forward a low-rank approximation algorithm of bilinear pooling, which is easy to operate and very effective. Yu et al. [18] had proposed the Multimodal Factorized Bilinear (MFB) Pooling and Multimodal Factorized High-order (MFH) [36] Pooling, which have achieved better performance. Reference [37] expanded the self-attention model for single modality into a unified attention model, which can describe the complex intra- and inter-modal interactions of multimodal data, generating excellent results.

## 3. Co-Attention Layer

### 3.1. Scaled Dot-Product Attention

The input of the scaled dot-product attention [21] includes queries, values, and keys of dimension $d_{k}$. It should be noted that the value vector and the key vector have the same dimension. The values, keys, and queries are packed into matrixes *V*, *K*, and *Q*, respectively. The attention function on all queries is performed simultaneously. The attended feature *F* is given by:

### 3.2. Multi-Head Attention

To further enhance the representation capacity of the attended features, multi-head attention is presented in Reference [25]. Multi-head attention is to perform multiple attention operations, which is composed of *h* paralleled heads, and each head corresponds to a scaled dot-product attention function. On each projection of the values, keys, and queries, the attention function is executed in parallel, resulting in output values of dimension $d_{v}$. Concatenate these attention functions to obtain the final attended features, as shown in Formulas (2) and (3):

(1) $$\begin{array}{c} MHAtt\left(Q,K,V\right)=Concat\left(head_{1},\ldots ,head_{h}\right)W^{O} \end{array}$$

(2) $$head_{i}=Att\left(QW_{i}^{Q},KW_{i}^{K},VW_{i}^{V}\right),$$

where $W_{i}^{Q}$, $W_{i}^{K}$, and $W_{i}^{V}$ are the projection matrices of the i-th head, and $W^{O}$ is the learned weight matrix. In this calculation, *h* = 8 parallel heads are applied to reduce each head’s dimensionality, and the total calculation consumption is the same as that of full-dimensional single-head attention. Additionally, $d_{model}$ = 512 is the dimensionality of the embedding layer. In each head:

(3) $$d_{k}=d_{v}=\frac{d_{model}}{h}.$$

### 3.3. Pointwise Feed Forward Layer

The pointwise feed-forward layer is a forward neural network, which uses several weight coefficients $W_{i}$, and the biased variable $b_{i}$ to perform linear operations and activation operations. It realizes the transformation of the output features through two fully connected layers with a ReLU activation and dropout. The Relu activation function makes the output of some neurons zero, which makes the neural network sparse, reduces the interdependence of parameters, and relieves the occurrence of the over-fitting problem. Suppose the input feature set is $E=\left[e_{1},\ldots ,e_{n}\right]\;\in R^{n\times d_{e}}$, the output can be written as:

(4) $$FFN\left(E\right)=\max\left(0,EW_{1}+b_{1}\right)W_{2}+b_{2}.$$

### 3.4. Self-Attention and Guided-Attention

#### 3.4.1. Self-Attention Unit

**Figure 1:**
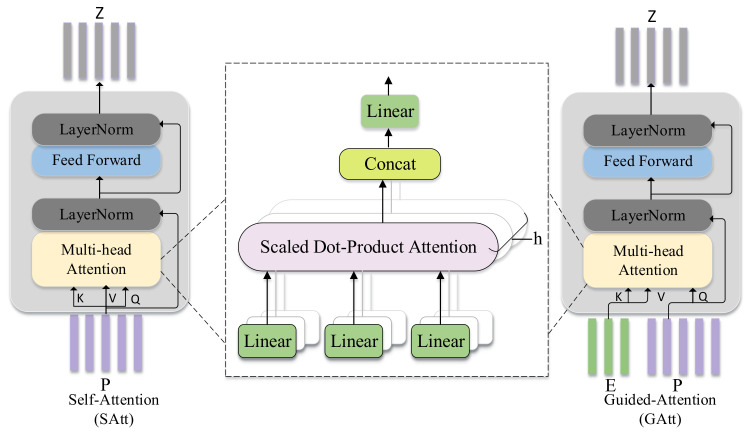
The composition of two basic components. The self-attention unit takes image features or question features as input, and the output feature is Z; the guided-attention unit adopts image features and question features as input, where image features are guided by the question features, and Z represents the output feature.

Both Self-Attention (SAtt) unit [25] and Guided-Attention (GAtt) unit [25] are based on multi-head attention. The self-attention unit takes question features or image features as input, which means question self-attention or image self-attention. As shown on the left side in Figure 1, the self-attention unit consists of the multi-head attention layer and pointwise feed-forward layer. In this paper, *E* and *P* represent question features and image features respectively. The input feature $P=\left[p_{1},p_{2},\ldots ,p_{m}\right]$ is transformed into three matrices: query matrix $Q_{P}$, key matrix $K_{P}$, and value matrix $V_{P}$. In the self-attention unit, the multi-head attention layer calculates the pairwise relationship between each region pair $\langle p_{i},p_{j}\rangle$ within an image. The attended output features $F_{1}$ can be expressed as:

(5) $$\begin{array}{cc} F_{1} & =MHAtt\left(Q_{P},K_{P},V_{P}\right)\\ & =Concat\left(head_{1},\ldots ,head_{h}\right)W^{o}, \end{array}$$

(6) $$head_{i}=Att\left(Q_{P}W_{i}^{Q_{P}},K_{P}W_{i}^{K_{P}},V_{P}W_{i}^{V_{P}}\right),$$

where $W_{i}^{Q_{P}}$, $W_{i}^{K_{P}}$, $W_{i}^{V_{P}}$ are the projection matrices of *i*-th head concerning image features. The feed-forward layer transforms the attended image features further. The final feature is obtained as follows:

(7) $$FFN\left(F_{1}\right)=\max\left(0,F_{1}W_{1}+b_{1}\right)W_{2}+b_{2},$$

where $W_{i}$ and $b_{i}$ represent weight coefficients and biased variable respectively.

#### 3.4.2. Guided-Attention Unit

Guided-attention unit takes the question features and image features as input, which represents question-guided attention or image-guided attention. Correspondingly, the output feature represents the image features guided by the question or the question features guided by the image. As shown on the right side in Figure 1, the image feature is guided by the question feature. The question can help to understand the image better and capture important image regions relevant to the question. The input features $P=\left[p_{1},p_{2},\ldots ,p_{m}\right]$ and $E=\left[e_{1},e_{2},\ldots ,e_{n}\right]$ are transformed into three matrices: query matrix $Q_{P}$, key matrix $K_{E}$, and value matrix $V_{E}$. In the guided-attention unit, the multi-head attention layer models the pairwise relationship between each pair $\langle p_{i},e_{j}\rangle$ from image and question. The attended feature $F_{2}$ is described as follows:

(8) $$\begin{array}{cc} F_{2} & =MHAtt\left(Q_{P},K_{E},V_{E}\right)\\ & =Concat\left(head_{1},\ldots ,head_{h}\right)W^{o} \end{array}$$

(9) $$head_{i}=Att\left(Q_{P}W_{i}^{Q_{P}},K_{E}W_{i}^{K_{E}},V_{E}W_{i}^{V_{E}}\right).$$

Input feature $F_{2}$ to the feed-forward layer:

(10) $$FFN\left(F_{2}\right)=\max\left(0,F_{2}W_{1}+b_{1}\right)W_{2}+b_{2}.$$

(11) $$F=Att\left(Q,K,V\right)=softmax\left(\frac{QK^{T}}{\sqrt{d_{k}}}\right)V,$$

where the softmax function is a generalization of logistic function and represents normalization. $Att\left(\cdot \right)$ represents an attention function, which is essentially the same as dot-product attention. It has two significant advantages in taking up less space and having a higher speed.

## 4. Network Architecture for Vqa

**Figure 2:**
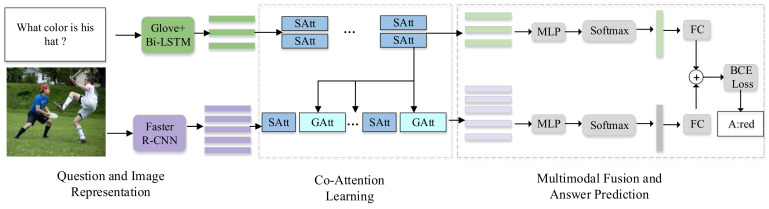
Overall flowchart of the improved dense multimodal co-attention network.

This section demonstrates DCAN in detail, the main structure of which is shown in Figure 2. Firstly, the initial feature representation of the question and image is described, then the dense multimodal co-attention model is presented. Finally, multimodal fusion and answer prediction are provided.

### 4.1. Feature Extraction

#### 4.1.1. Question and Answer Representation

**Figure 3:**
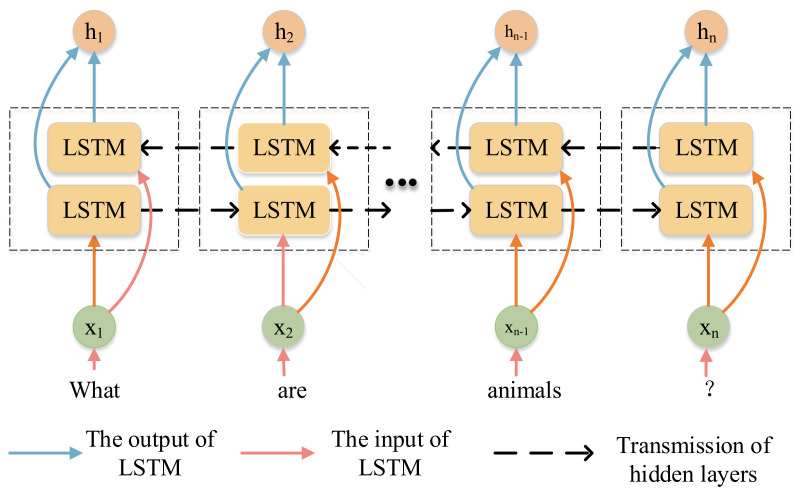
Structure of bidirectional long short term memory (LSTM).

The questions and answers are encoded by Bi-LSTM. In Figure 3, the network structure of Bi-LSTM is shown. The question is tokenized and divided into words with a maximum of 14, and the excess is left out. Each word will be transformed into a vector representation and pre-trained by Glove [38]. Specifically, a question is first transformed into a sequence $\left\{w_{1}^{Q},\ldots ,w_{n}^{Q}\right\}$, and then input into Bi-LSTM with the residual connection.

(12) $$\vec{q_{n}}=\mathrm{Bi}-\mathrm{LSTM}\left(\vec{q_{n-1}},w_{n}^{Q}\right)$$

(13) $$\overset{\leftarrow }{q_{n}}=\mathrm{Bi}-\mathrm{LSTM}\left(\overset{\leftarrow }{q_{n+1}},w_{n}^{Q}\right),$$

where $\vec{q_{n}}$ is the output value of the forward hidden layer, and $\overset{\leftarrow }{q_{n}}$ is the output value of the backward hidden layer.

It is assumed that $Q=\left[q_{1},\ldots ,q_{N}\right]\in R^{d\times N}$ is the feature representation matrix of the question, where $q_{n}={\left[{\vec{q_{n}}}^{T},{\overset{\leftarrow }{q_{n}}}^{T}\right]}^{T}\;\left(n=1,\ldots ,N\right)$. We use $S_{Q}={\left[{\vec{q_{N}}}^{T},{\overset{\leftarrow }{q_{1}}}^{T}\right]}^{T}$ to connect the last hidden states in the forward and backward paths, where $\vec{q_{N}}$ is the final output of the forward hidden layer, and $\overset{\leftarrow }{q_{1}}$ is the final output of the backward hidden layer.

When encoding the answers, a similar method as the question encoding method is adopted. Supposing that an answer has words with the number of *M*, it can be encoded as $\left\{w_{1}^{A},\ldots ,w_{M}^{A}\right\}$ and then inputted to the same Bi-LSTM, resulting in the hidden states $\vec{a_{M}}$ and $\overset{\leftarrow }{a_{1}}$. We use $S_{A}={\left[{\vec{a_{M}}}^{T},{\overset{\leftarrow }{a_{1}}}^{T}\right]}^{T}$ to connect the last hidden states in the forward and backward paths.

#### 4.1.2. Image Representation

Inspired by bottom-up attention [31], Faster R-CNN in conjunction with ResNet-101 CNN [39] is used to obtain the target-level image representation. Faster R-CNN is an object detection model used to identify object regions about specific classes and localize them with bounding boxes. It is mainly composed of two modules: Region Proposal Network (RPN) and the detection module. It can be further divided into four parts: convolution layers, RPN, RoI (Region of Interest) pooling, classification and regression.

The output feature is $P\in R^{c\times d}$, where $c\in \left[10,100\right]$ denotes the total number of object detection features, and *d* represents the dimensionality of each feature in each image. Considering better performance, lower cost and computational efficiency, c=36 is set.

### 4.2. Dense Co-Attention Model

**Figure 4:**
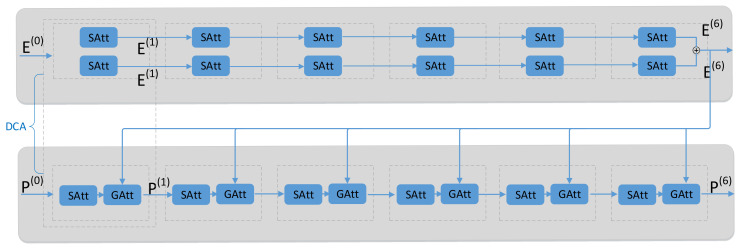
Dense multimodal co-attention model. ⊕ denotes adding up the question features.

As can be seen in Figure 4, the dense co-attention model consists of six DCA layers. In other words, six layers of SAtt (E)-SGAtt (P, E+E) are stacked to realize the dense intra- and inter-modal interactions. Each DCA layer contains two parallel question self-attention units, an image self-attention unit, and a question-guided unit. The process of dense co-attention learning is defined as follows:

Firstly, taking the original question features $E^{\left(0\right)}$ as input and output $E^{\left(1\right)}$ through a layer of the self-attention unit. For each SAtt unit, the input of each layer is the output of the previous layer. It can be defined as follows:

(14) $$E^{\left(t\right)}=SAtt\left(E^{\left(t-1\right)}\right),$$

where $t\in \left[1,6\right]$; add up the question features obtained from the two parallel question self-attention units, and then input them into the subsequent guided-attention unit to guide the image.

Secondly, the original image features are input to a layer of the self-attention unit to model self-attention of the image. Then the obtained image features are fed to the guided-attention unit together with the question features in the above step. For each SGAtt unit, the output feature of each layer is defined as Equation (15):

(15) $$P^{\left(t\right)}=SGAtt\left(P^{\left(t\right)},E^{\left(L\right)}+E^{\left(L\right)},\right)$$

where $t\in \left[1,6\right]$; the number of DCA layers *L* is set to 6. SGAtt means the image self-attention is carried out firstly, then the question-guided attention is performed.

### 4.3. Multimodal Fusion and Answer Prediction

After co-attention learning, the question features and image features contain abundant information about the attention weights of words and regions. Therefore, a two-layer multi-layer perceptron (MLP) is designed as an attention reduction model, which can obtain the attended features of both the question and the image. If the image feature *P* is taken as an example, the final attended feature $\bar{P}$ can be expressed as follows:

(16) $$\lambda =softmax\left(MLP\left(P^{\left(L\right)}\right)\right)$$

(17) $$\bar{P}=\sum_{j=1}^{n}\lambda_{j}{p_{j}}^{\left(L\right)},$$

where $\lambda =\left[\lambda_{1},\ldots ,\lambda_{n}\right]\in R^{n}$ is the learned weight, and *L* is the number of layers stacked by DCA layers, namely L=6. The softmax function is used to standardize the weights of attention on all regions. Then, image features from all regions are weighted and added into a single vector $\bar{P}$ as the representation of image features.

After calculating the final image features $\bar{P}$ and text features $\bar{E}$, they are fused with linear multimodal fusion function. The fused feature is expressed by Formula (18):

(18) $$C=LayerNorm\left(W_{E}^{T}\bar{E}+W_{P}^{T}\bar{P}\right),$$

where *C* is the joint representation of question and image. In this paper, *C* is input into a non-linear layer, and the score of each candidate answer is predicted by linear mapping.

(19) $$s=sigmoid\left(W_{0}Relu\left(W_{f}C\right)\right),$$

where *s* is the score of the candidate answer, $W_{0}$ and $W_{f}$ are linear projection matrix. The most popular approach to answer prediction is to model answer prediction as a classification problem. Firstly, the most common answers are selected to form the answer candidate sets according to the training set. Then by seeing each candidate answer as a class, the probability distribution of the correct answer on the answer candidate set is predicted. Finally, the candidate answer with the highest probability is selected as the prediction result.

The binary cross-entropy (BCE) is employed as the loss function to train the classifier of N answers.

(20) $$L=\sum_{i=1}^{N}\gamma_{i}\log\left(s_{i}\right)+\;\left(1-\gamma_{i}\right)\log\left(1-s_{i}\right),$$

where $r\in R^{N}$ represents the matching degree between the question and prediction.

## 5. Experiments and Results

In this section, DCAN is evaluated on the VQA-v2 dataset. Firstly, the dataset is introduced, and then experimental demonstrations and results are highlighted. Finally, the qualitative analysis is presented.

### 5.1. Dataset

The VQA-v2 dataset is based on MSCOCO [40], which contains 1,105,904 questions raised by humans and 204,721 images from the COCO dataset. The dataset can be divided into 40%, 20%, and 40% for the training set, validation set, and test set. All the questions are divided into three categories: Yes/No, Number, and Others. Compared with the VQA-v1 dataset, VQA-v2 collects more samples. Besides, the more balanced VQA-v2 can cope with the possibility of accuracy improvement caused by overfitting. It emphasizes visual understanding by reducing text deviation. Specifically, each question in the dataset corresponds to two images, so that each question has two different answers.

### 5.2. Experimental Setup

The question feature $E\in R^{14\times 512}$ is extracted with one-layer Bi-LSTM, and the number of nodes in the hidden layer is set to 512. Images are expressed as a collection of 36 local areas by using bottom-up and top-down attention. To train DCAN, we use Adam solver with $\beta_{1}$ = 0.9 and $\beta_{2}$ = 0.99. Since the large-scale Visual Genome is used to augment the training set in this paper, training is stopped at 200,000 iterations. To predict the answer, we use the most common *N* answers as *N* classes and set the number of answers to 3000. The dropout ratio in each fully connected layer is set to 0.1 to prevent overfitting. Due to GPU memory limitation, the batch size of the model is set to 64, and 13 epochs of training are performed. Finally, the best epoch is chosen in the validation set.

### 5.3. Ablation Analysis

In this section, some ablation experiments are conducted on the VQA-v2 dataset to verify the effectiveness of DCAN. For a fair comparison, all models use bottom-up object features, which are extracted from Faster R-CNN. The ablation studies are trained on the train set to save the training time, and the results are evaluated on the validation set.

#### 5.3.1. Effectiveness of Dca

As shown in Table 1, we conduct ablation studies to explore the effectiveness of different attention models. ID(E)-GAtt (P, E) denotes taking the original question features as input, and modeling question-guided image attention. SAtt(E)-GAtt (P, E) means question self-attention and question-guided attention. It can be seen that SAtt(E)-GAtt (P, E) outperforms ID(E)-GAtt (P, E), which proves that it is beneficial to set self-attention for questions. Besides, the result of SAtt(E)-SGAtt(P, E+E) is better than that of SAtt(E)-SGAtt(P, E), which indicates that compared with the single-layer question self-attention unit, two parallel self-attention units can extract more fine-grained question features. When the more fine-grained question features to guide the image, it can provide supplementary and rich information to help better understand the image, facilitating more accurate multimodal reasoning, thus improve the performance of VQA.

#### 5.3.2. Number of Heads

To explore the effect of the number of heads in multi-head attention on the accuracy, we set the number of heads h∈{2,4,8,16}. In our best model, the default number of heads is set to 8. As shown in Table 1, the accuracy of the model also continues to improve as the number of head increases. When *h* is 16, accuracy is no longer improved. Considering the training time, we set *h* = 8 in our bes model.

#### 5.3.3. Question Representation

As shown in Table 1, the effectiveness of Bi-LSTM is explored under one DCA layer, which shows that the performance of Bi-LSTM is slightly better than that of LSTM. The reason is that it can capture rich semantic information during the question encoding phase, which is beneficial to feature extraction in the dense co-attention learning module. Therefore, Bi-LSTM is adopted to encode questions in this paper.

#### 5.3.4. Depth of DCA

To explore the effect of the depth of DCA on the accuracy, we set the number of DCA layer L∈{2,4,6,8}. As can be seen from the results in Table 2, as the number of stacked DCA layers increases, the accuracy of the model also continues to improve. The attention of the model gradually focuses on the most critical regions. It will eventually approach saturation, so it can be seen that the improvement is no longer evident from the eighth layer. Considering the overall efficiency of the model, we set the depth of DCA to 6.

### 5.4. Comparisons with Existing Methods

In this section, DCAN is compared with state-of-the-art methods under the same experimental settings. We use the train set, vg set, and validation set to train all models, where vg represents the augmented training samples from Visual Genome. Table 3 has two parts, which shows the results of the comparison with the latest methods. The first part is the results of its comparison with other attention models. The second part shows the results of its comparison with the state-of-the-art method MCAN and MCAN is regarded as the baseline of this paper.

First of all, the first part of Table 3 is the results of its comparison with other attention models. Among them, Bottom-up is the winner of the VQA challenge 2017 and is the first to employ detected object features instead of grid features. MFH presents a generalized multimodal factorized high-order pooling by cascading multiple MFB modules. BAN uses bilinear interactions to make the most use of visual and text information. BAN + counter means introducing the counting mechanism based on the BAN network architecture. The core of DCN is to improve the fusion ability of vision and language by the dense symmetric interaction between question and image. Reference [41] proposes a new framework for dynamic fusion with intra- and inter-modality. MCAN consists of a cascade of modular co-attention layers.

It can be seen from Table 3 that the approach proposed in this paper outperforms BAN, MFH, and DCN by a large margin of 1.37%, 2.13%, and 4.02%, respectively. The prime reason is that they neglect the dense self-attention in each modality, which in turn shows the importance of self-attention modeling. In terms of the overall accuracy, our network is 0.67% higher than DFAF. The reason is that DFAF learns redundant question features during the intra-modality interaction. Since the information inside image features are dynamically conditioned on the question features, irrelevant image features are acquired.

**Figure 5:**
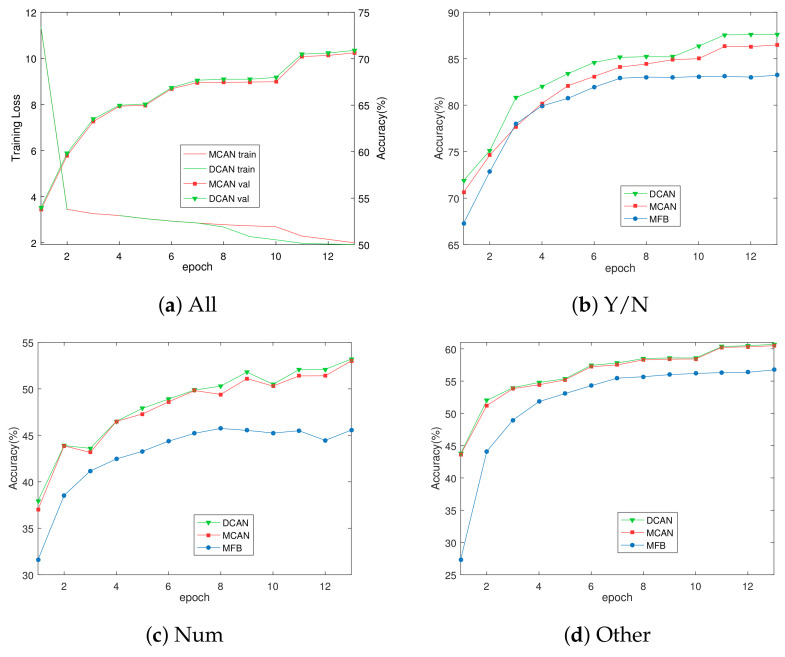
(**a**) The training loss and validation accuracy vs. epoch of MCAN and DCAN. BCE loss is used for all methods; (**b**–**d**) the overall and per-type accuracies of DCAN, MCAN, and MFB.

Secondly, to further verify the effectiveness of DCAN, the second part of Table 3 shows the results based on its comparison with MCAN, the champion of the VQA challenge in 2019. It is observed that the proposed approach outperforms MCAN by a large margin of 0.26 and 0.31 points on both test-dev and test-standard sets. It is worth noting that the improvements can be seen in all of the entries (Yes/No with 1.2%, Number with 0.14%, Other with 0.16%). The reason is that in MCAN, a single-layer self-attention unit is used to learn the relationship between words in the question. While in DCAN, more fine-grained question features can be obtained by adding up the features obtained by performing question self-attention twice. The image is guided by the question, thus resulting in more accurate image features. Besides, Figure 5 shows the validation course of 13 epochs, from which it can be seen that the accuracy of DCAN on the validation set is far better than that of MCAN and MFB in every epoch. Moreover, since the seventh epoch, the loss value decreases faster than MCAN, which indicates that DCAN has a stronger learning ability.

### 5.5. Qualitative Analysis

**Figure 6:**
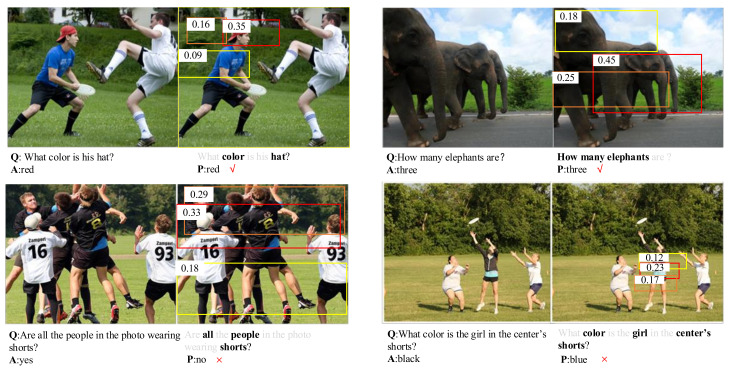
Typical examples of the learned image and question attention. The (**top row**) shows two examples of the correct prediction while the (**bottom row**) shows two incorrect predictions. For each example, the image, question (Q), answer (A) and prediction (P) are displayed in turn, followed by the learned image and question attentions. The brightness of the text and the score of the object proposal box represent the corresponding attention weight.

In this section, some results of the DCAN are visualized in Figure 6. Four examples are given, which are randomly selected from the validation set. The first row shows two examples of successful predictions, while the second row shows two incorrect predictions. The brightness of the text and the probability value of the object proposal box represent their importance in the attention weights. The probability value of the attention is shown on the top left corner of each bounding box. The larger the probability value, the higher the corresponding attention weight. In the first row, it can be seen that DCAN accurately locates the most relevant object proposal box, and then outputs the corresponding score. The red object bounding box corresponds to the highest probability. It can be seen from the left side of the second row that six people are catching something. The more relevant the word in question is to the image, the brighter the word is, so the words “all”, “people”, and “shorts” are highlighted. For image attention, the red bounding box has the highest attention probability of 0.33, but it does not include everyone. The prediction is “no”, which is not consistent with the correct answer.

## 6. Conclusions

This paper focuses on fine-grained interactions between multimodalities in VQA tasks. An effective Dense Co-attention Networks (DCAN) for the VQA task is developed, the core of which is a dense co-attention model. It consists of six layers of self-attention units and guided-attention units, namely, six layers of SAtt (E)-SGAtt (P, E+E), which achieves the fine-grained and simultaneous understanding of both images and questions. Moreover, to better capture the relationship between words that are relatively far apart and make the extracted semantics more robust, Bi-LSTM is adopted in the question encoding phase to encode the bidirectional semantic features of the question. Compared with the existing method MCAN, DCAN can make use of the complex correlation between multimodal features in a more effective way and extract more discriminative features for images and questions. This exploration of modeling dense intra- and inter-modality interactions has been applied to intelligent transportation [42], intelligent robot [43], and other fields [44,45,46]. Applying it to a wider range of scenarios will be an inevitable trend in the future.

## Figures and Tables

**Table 1:** Ablation studies of the question encoding, the single-layer attention model, and the number of heads on Visual Question Answering (VQA)-v2 Validation set.

Module	Setting	Accuracy
Question encoding	Bi-LSTM	65.71
	LSTM	65.6
Number of heads	*h* = 2	65.38
	*h* = 4	65.51
	*h* = 8	65.67
	*h* = 16	65.67
Attention model	ID(E)-GAtt(P, E)	64.8
	SAtt(E)-GAtt (P, E)	65.2
	SAtt(E)-SGAtt (P, E)	65.4
	SAtt(E)-SGAtt (P, E+E)	65.6

**Table 2:** Ablation studies of the number of Dense Co-Attention (DCA) layer *L* on VQA-V2 validation set, where L∈{2,4,6,8}.

L	Y/N	Number	Other	All
2	84.36	47.98	57.92	66.55
4	84.74	49.01	58.37	67.05
6	84.96	49.20	58.30	67.13
8	84.93	49.45	58.28	67.14

**Table 3:** Comparison with the state-of-the-art methods on the VQA-v2 dataset.

Model	Test-Dev				Test-Std
	All	Y/N	Num	Other	All
Bottom-up [31]	65.32	81.82	44.21	56.05	65.67
MFH [36]	68.76	84.27	49.56	59.89	-
BAN [19]	69.52	85.31	50.93	60.26	-
BAN+counter [19]	70.04	85.42	54.04	60.52	70.35
DCN [20]	66.87	83.51	46.61	57.26	-
DFAF [41]	70.22	86.09	53.32	60.49	70.34
MCAN [25]	70.63	86.82	53.26	60.72	70.9
DCAN (ours)	**70.89**	**88.02**	**53.40**	**60.88**	**71.21**

## Abbreviations

The following abbreviations are used in this manuscript:

VQA	Visual Question Answering
Bi-LSTM	Bidirectional Long Short-Term Memory
CNN	Convolutional neural network
RPN	Region Proposal Network
ROI	Region of Interest
MFB	Multimodal Factorized Bilinear
MFH	Multimodal Factorized High-order
BAN	Bilinear Attention Networks
AI	Artificial Intelligence
